# Effect of resveratrol on alcohol-induced mortality and liver lesions in mice

**DOI:** 10.1186/1471-230X-6-35

**Published:** 2006-11-14

**Authors:** Luis Bujanda, María García-Barcina, Virginia Gutiérrez-de Juan, Joseba Bidaurrazaga, Marian Fernández de Luco, Marian Gutiérrez-Stampa, Mikel Larzabal, Elisabeth Hijona, Cristina Sarasqueta, Miguel Echenique-Elizondo, Juan I Arenas

**Affiliations:** 1 Department of Gastroenterology, Donostia Hospital, San Sebastián, Spain; 2 Department of Genetics, Basurto Hospital, Bilbao, Spain; 3 Department of Cellular Biology, Basque Country University, Spain; 4 Department of Pathology, Cruces Hospital, Baracaldo, Spain; 5 Department of Pathology, Donostia Hospital, Spain; 6 Department of Surgery, Basque Country University, San Sebastián, Spain

## Abstract

**Background:**

Resveratrol is a polyphenol with important antiinflammatory and antioxidant properties. We investigated the effect of resveratrol on alcohol-induced mortality and liver lesions in mice.

**Methods:**

Mice were randomly distributed into four groups (control, resveratrol-treated control, alcohol and resveratrol-treated alcohol). Chronic alcohol intoxication was induced by progressively administering alcohol in drinking water up to 40% v/v. The mice administered resveratrol received 10 mg/ml in drinking water. The animals had free access to standard diet. Blood levels were determined for transaminases, IL-1 and TNF-α. A histological evaluation was made of liver damage, and survival among the animals was recorded.

**Results:**

Transaminase concentration was significantly higher in the alcohol group than in the rest of the groups (p < 0.05). IL-1 levels were significantly reduced in the alcohol plus resveratrol group compared with the alcohol group (p < 0.05). TNF-α was not detected in any group. Histologically, the liver lesions were more severe in the alcohol group, though no significant differences between groups were observed. Mortality in the alcohol group was 78% in the seventh week, versus 22% in the alcohol plus resveratrol group (p < 0.001). All mice in the alcohol group died before the ninth week.

**Conclusion:**

The results obtained suggest that resveratrol reduces mortality and liver damage in mice.

## Background

Alcohol abuse is a major cause of liver disease in Western countries. In the United States it has been estimated that there are some 25,000 deaths from cirrhosis yearly, about half of which are related with alcohol [1]. The cirrhosis death rate (irrespective of cause) in the United States is about 12 per 100,000 population, while the 5 and 10-year survival rates for alcoholic cirrhosis are reported at 23% and 7%, respectively, much worse than other types of cirrhosis [1].

Alcohol can cause liver damage in the form of steatosis or fatty liver, hepatitis, fibrosis and liver cirrhosis. In general, the amount and duration of alcohol abuse correlate with the presence and severity of liver damage, at least as regards the initial stage of fatty liver [2]. It is still uncertain, however, why only 20–30% of long-term alcohol abusers develop severe liver disease. For instance, one study estimated that cirrhosis develops in only 14% of alcoholics consuming 160 g of alcohol (12 drinks) per day for 8 years [1]. The relatively low incidence of severe liver damage in alcoholics and the poor correlation between the amount of alcohol and the severity of the disease suggest that in addition to genetic influences, the pathogenesis of alcoholic liver disease involves other important environmental factors [3-5].

One hypothesis is that substances such as polyphenols may be present in the diet, or even in alcoholic beverages, thereby contributing to inhibit alcohol induced damage. Polyphenols possess a variety of biological functions, including antioxidant, anti-inflammatory, and anticancer effects. Resveratrol is a phytoalexin polyphenolic compound found in various plants, including grapes, berries, and peanuts. Multiple lines of compelling evidence indicate its beneficial effects on neurological, hepatic, and cardiovascular systems. Resveratrol has also been reported to have anti-cancer, anti-mycotic, and anti-bacterial properties [6-9].

Among the possible mechanisms responsible for its biological activities are downregulation of the inflammatory response through inhibition of synthesis and release of pro-inflammatory mediators, modification of eicosanoid synthesis, inhibition of Kupffer cells and adhesion molecules, inhibition of activated immune cells, or inhibition of inducible nitric oxide synthase (iNOS) and cyclooxygenase-2 (COX-2) via its inhibitory effects on nuclear factor (kappa)B (NF-(kappa)B) or the activator protein-1 (AP-1) [5,10-12]. Pharmacokinetic studies revealed that the target organs of resveratrol are liver and kidney, where it is concentrated after absorption and is mainly converted to a sulfated form and a glucuronide conjugate [13].

The present study investigates whether resveratrol is able to reduce alcohol-induced mortality and liver damage in mice.

## Methods

### Animals and diets

Male Balb/c mice (CRIFFA, Barcelona, Spain) weighing about 26 g were used. All experiments were conducted in accordance with the Guide for the Care and Use of Laboratory Animals published by the US Public Health Service. Animals were maintained on a regular 12-hour light period at a controlled temperature (25 ± 2°C), with free access to food and water. The mice were adapted for 2 to 5 days prior to initiation of the experimental protocol. The diet consisted of 58.5% carbohydrates, 15.5% proteins, 2.7% fat, 5.5% minerals, 3.7% fiber and 12% humidity (PANLAB, Barcelona, Spain). The caloric contents were 3000 kcal/kg.

### Experimental procedures

Mice were distributed into four groups: control, resveratrol-treated control, alcohol and resveratrol-treated alcohol. Resveratrol was obtained from SIGMA Chemical, (Pool, Dorset) and alcohol from PRS.PANREAC. In order to induce alcoholic intoxication, pure alcohol was progressively diluted in the drinking water: 10% (v/v) alcohol in the first week, 20% in the second, 30% in the third, and 40% in the fourth and subsequent weeks until the end of the study. Resveratrol (10 mg/ml) was also added to the water. Liquids and food were changed twice a week and the animals were monitored daily for general health.

The timing of sacrifice for the study of liver damage was determined from previous trials in which mortality was seen to be very high after the sixth week. The animals were killed at 6 weeks using intraperitoneal pentobarbital (Nembutol). The animals destined for the evaluation of mortality were followed until death.

The mortality and liver-damage studies were each repeated on three occasions to confirm the histological and laboratory alterations (6 mice per group, for a total of 18 animals per group) and again on three occasions to confirm the mortality curves (6 mice per group, for a total of 18 animals per group).

### Laboratory tests

At the time of sacrifice alcohol in blood was determined by the AxSYM REA assay, a quantitative reagent system for the measurement of ethanol in murine whole blood (Abbott Axsym System). Laboratory parameters such as AST, ALT, creatinine, urea, total cholesterol, triglycerides, GGT, amylases, total bilirubin and hematocrit were determined on a computer-controlled biochemical analyzer.

### Determination of IL-1 and TNF-α

Serum TNF-α and IL-1β concentrations were measured using ELISA kits based on anti-mouse TNF-α and IL-1β monoclonal antibodies (R&D Systems, Boston, MA).

### Liver histology

The histological study was conducted following a midline laparotomy to remove the liver. Samples of liver tissue removed at the time the mouse was killed were placed immediately in 10% buffered formalin and subsequently embedded in paraffin. Sections were stained with hematoxylin and eosin using standard techniques. Sections were viewed without knowledge of the treatment group to which each animal had belonged.

### Statistical analysis

Quantitative data were expressed as an average ± SD for the different animals in each group, and comparisons were made using the 2-tailed Student's *t *test. P values of < 0.05 were considered statistically significant.

## Results

### Mortality

The mortality curves were similar in all three test series.

**Figure 1:**
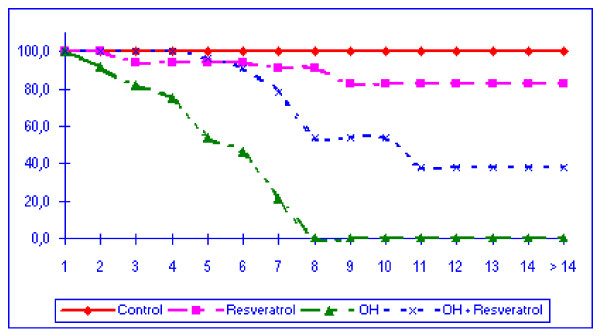
Survival of the different groups of mice over time (weeks). Mortality was of 100% at 8 weeks in the alcohol (OH) group and only 50% in the (OH-Resveratrol) group. (OH; alcohol group).

The mice in the alcohol group began to die after the second week of alcohol intoxication, with a survival of 22% (4/18) in the seventh week. None of the mice survived beyond eight weeks. The animals belonging to the alcohol plus resveratrol group began to die later (after the fourth week, involving a single mouse), with a survival of 78% (14/18) in the seventh week. The control and resveratrol groups in turn presented survivals of 100% and 89% (16/18), respectively, in the seventh week (Figure 1). Survival was significantly lower in the alcohol group than in the other three groups (p < 0.001).

The mice subjected to alcohol intoxication showed a poorer general condition after the second week, as reflected by decreased activity, immobility, grouping and coarse hair. No such differences were observed in the other three groups (control, resveratrol and alcohol plus resveratrol).

### Food and water intake

The average food intake among the control rats was 4.27 ± 0.86 g/day, which was similar to the consumption in the resveratrol group (4.47 ± 0.63 g/day). The alcohol group and the alcohol plus resveratrol group showed an important reduction in food intake, with 3.28 ± 0.86 and 3.11 ± 0.51 g/day, respectively. These differences between the group with alcohol and the groups without alcohol were statistically significant (p < 0.05).

Similar observations applied to liquid consumption, which on average was similar in the control group and in the resveratrol group (5.98 ± 2.07 ml/day and 5.50 ± 0.10 ml/day, respectively), while the liquid consumed in the alcohol groups was comparatively less (3.14 ± 1.46 ml/day in the alcohol group and 2.97 ± 0.97 ml/day in the alcohol plus resveratrol group)(p < 0.05). Each mouse on the alcohol and alcohol plus resveratrol groups did ingested approximately 3 ml, that is, around 30 mg of resveratrol and 0.24 g of alcohol per day.

**Figure 2:**
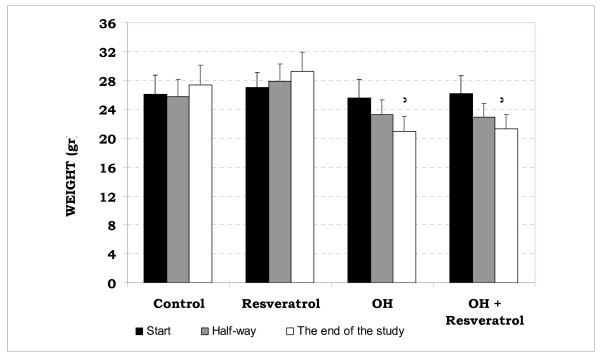
Body weights of the mice at the start, half-way and at the end of the study. Weight increase was observed in the control and resveratrol groups while a decrease existed in the alcohol group. Weight decrease was less evident in the OH-Resveratrol group. *P *< 0.05 vs. control group and resveratrol group. (OH; alcohol group).

Body weight in the control and resveratrol group was maintained during the study, while the corresponding values in the alcohol groups were seen to decrease (p < 0.05)(Figure 2). The drop in body weight was more pronounced in the group administered alcohol only, although there were no significant differences compared with the alcohol plus resveratrol group.

### Laboratory findings

**Figure 3:**
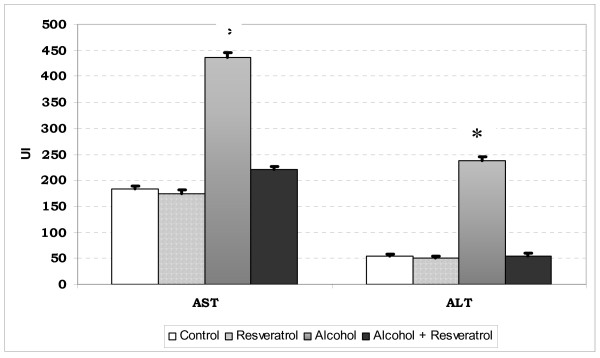
AST and ALT plasma levels in each group are shown. * *P *< 0.05 vs. other groups.

ALT concentration in the alcohol group was significantly higher than in the rest of the groups (p < 0.001). AST concentration in the alcohol group was significantly higher than in the alcohol plus resveratrol group (p < 0.05). Stastiscally significant differences in AST levels were also observed between the two groups with alcohol and the two groups without alcohol (p < 0.05) (Fig. 3).

The AST/ALT ratio in the control series and resveratrol group was 3.4 and 3.5, respectively. In contrast, the ratio in the alcohol group and alcohol plus resveratrol group was 1.87 and 4.16, respectively.

The values for alkaline phosphatase (AP) were 208.5 ± 16.5 U/l in the control group and 209 ± 14 U/l in the resveratrol group. AP in the alcohol plus resveratrol group was higher (262 ± 17 U/l) than in the alcohol group (237.5 ± 15 U/l)(p = 0,5).

There were no differences in terms of the other laboratory parameters such as creatinine, urea, total cholesterol, triglycerides, GGT, amylases, total bilirubin and hematocrit.

Arterial blood alcohol determinations revealed a mean value of 34.73 mg/dl (range 15.52 – 55.45 mg/dl) among the mice administered alcohol in water solution, while no alcohol was detected in the blood in any of the control or resveratrol groups.

### Interleukins

**Figure 4:**
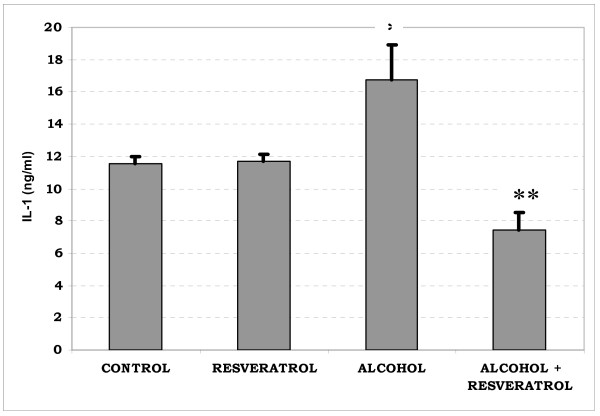
Serum concentrations of interleukin-1 (IL-1) in the different groups. The control group and resveratrol group showed higher plasma levels than those observed in the alcohol plus resveratrol group. * *P *< 0.05 vs. control group, Δ *P *< 0,001 vs alcohol group.

Figure 4 shows that the IL-1 levels were increased in the alcohol group versus the rest of the groups (p < 0.05). The blood levels of IL-1 in the alcohol plus resveratrol mice were even lower than in the control group and resveratrol-treated control group. The blood levels of IL-1 in the alcohol plus resveratrol mice were lower than in the alcohol group (p < 0,001). TNF-α was not detected in any group.

### Histological evaluation

**Figure 5:**
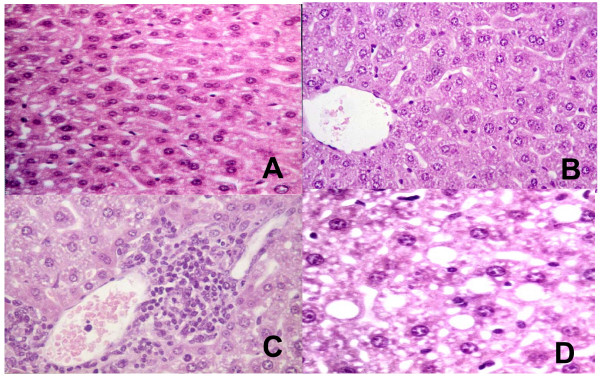
Liver sections from mice in control group (A) and resveratrol group (B) showed normal liver histology. Liver sections from a mouse in alcohol group showed mixed lympho-monocytic infiltrations around the portal vein (C). Mild steatosis with small fat droplets in the liver obtained from a mouse in alcohol group (D).

The histological liver lesions were mild in the alcohol and alcohol plus resveratrol groups compared with the laboratory alterations and critical condition of the mice. The observed lesions consisted of moderate mixed lympho-monocytic infiltrations with steatosis ranging from 10–15% (Figure 5). No fibrosis or liver cirrhosis was observed.

## Discussion

Alcohol is toxic for both humans and animals, and exerts important negative effects upon the liver, brain, heart, skeletal muscle, pancreas, hematological and immune systems, gastrointestinal apparatus and endocrine system [14,15]. Chronic alcohol abuse induces multiorgan deterioration with an increase in mortality. In humans, for example, alcoholics with severe denutrition suffer a 54% mortality rate after 12 months versus 9% among patients with mild denutrition [16]. The present experimental study found chronic (6 weeks in mice with an average lifespan of two years) and severe alcohol intoxication (40% of all fluid intake in the form of alcohol) to cause increased mortality after the second week. Food and alcohol intake was similar in the alcohol and alcohol plus resveratrol mice groups but a strong difference in mortality was observed between them. The cause of death was not known, though the most likely explanation is multiorgan failure secondary to denutrition, coagulation disorders (hypercoagulability was seen in the alcohol group, thus complicating blood extraction), liver alterations, increased infections and endotoxemia. Oxidative stress and endotoxin (lipopolysaccharide (LPS)) are commonly elevated in the blood of alcoholics and in certain animal models of alcoholic liver disease [17]. It causes the release of a battery of cytokines from macrophages and other cells. Reactive oxygen species are capable of damaging the cell in many ways. They have been shown to react with all major classes of cellular constituents: lipids, proteins and nucleic acids. It is well established that chronic ethanol administration leads to oxidative stress in the liver and other organs [18]. Thus, a high amount of alcohol intake, as observed in this model, has deleterious effects on circulatory, immune and neural systems as well as on blood elements, endocrine glands, pancreas, liver and muscles and bone leading to increased in mortality [19].

Alcoholic patients have a high prevalence of protein-calorie malnutrition and vitamin deficiencies [20,21]. In our study a significant decrease was observed in food ingestion and body weight among the alcohol-consuming mice. There are many reasons for malnutrition, perhaps the most important of which is decreased intake of nutrients. Calories derived from alcohol, are called "empty calories"., in reference to the fact that alcohol, which has 7.1 kcal/g, is not utilized properly by the body as a nutrient [22]. Thus, its intake is not associated with normal (expected) weight gain. The reasons for decreased intake of foods are many and include alcohol per se, presence of liver disease, impairment of the taste and smell acuity, maldigestion (decrease in bile salts or lipase), malabsorption (changes in gut mucosa and its enzymes, altered permeability, changes in motility and possibly gut edema) [23].

The alcohol plus resveratrol group showed an important decrease in mortality. The mechanisms by which resveratrol reduces alcohol-induced mortality are not clear, though its antioxidant, antiinflammatory and antiinfectious properties may play a role [5-7,10,23]. Resveratrol also suppresses the expression of inducible nitric oxide synthase and cyclooxygenase-2, is a potent inhibitor of nuclear factor kB (NF-kB) nuclear translocation by blocking kB kinase activity, and decreases platelet aggregation [24-28]. In our study, one mouse died in the resveratrol group. This death may have been due to aggression between individuals, as this mouse had paw lesions. In another published study involving resveratrol administered orally to mice at doses similar to our own, no increased mortality in the resveratrol group versus the controls was likewise observed [29].

The chronic consumption of high doses of alcohol can cause liver damage and cirrhosis in humans. However, in animals it is very difficult to establish an experimental model of alcoholic liver disease. Very few such models have been developed, and most do not involve only alcohol, i.e., they also include additional elements such as hypercaloric diets, iron, endotoxins or other toxins. The model closest to reality (animals with free access to food and drink) is that described by Tsukamoto et al. [30], though it induces alcoholic liver disease by administering a diet rich in polyunsaturated fats and alcohol through a nasogastric tube. Our own model attempts to reproduce situations as close as possibly to reality, i.e., the mice are able to spontaneously eat (standard diet) and drink as much as they want, without the influence of factors that enhance the liver damaging capacity of alcohol. The alcohol concentration in mouse arterial blood was modest, since alcohol consumption was irregular (i.e., free access). This may possibly be the reason why the blood alcohol concentrations and liver lesions in the mice were modest. The alcoholic intoxication model employed, and probably also the action of genetic factors, yields moderate liver damage without either liver fibrosis or cirrhosis – despite the high alcohol concentrations involved. In unpublished studies we found that liver cirrhosis could not be achieved in mice given lower doses of alcohol for longer periods of time (20% of drinking volume for 6 months).

Although cirrhosis could not be induced, the alcohol group showed a moderate increase in transaminase levels, with a greater rise in ALT, as occurs in alcoholic liver disease [31]. These increased laboratory values are not directly correlated to the histological severity of the disease as in humans [32]. The generation of free radicals as a result of alcohol did not correlate with elevated pathology scores in the liver or high ALT levels and acts as redox signals for cytokine production but does not directly damage the liver [33]. Resveratrol decreased the liver laboratory values and the corresponding liver damage – probably as a result of the diminished release of proinflammatory cytokines such as IL-1 as a result of Kupffer cell activation. In this context, over 75% of all individuals with alcoholic hepatitis have increased plasma IL-1 levels [34,35]. TNF-alfa serum levels have been related to mortality and the severity of the hepatic lesions observed in humans and produced by alcohol intake [35,36]. However TNF-alfa levels are poor diagnostic markers for the severity of TNF-dependent liver inflammation [37]. Other studies dealing with plasma levels of TNF-alfa and IL-1 in response (LPS), indicate that the TNF-alfa plasma level rises soon after the injection of LPS and that is the reason why it cannot be observed in our study. Otherwise IL-1 remains elevated for a longer period of time. This can be explained by augmented clearance of TNF-alfa not affecting IL-1, a smaller systemic inflammatory response or to neutralization of TNF-alfa by a soluble receptor resulting in a marked attenuation of the late phase of the alcohol action [38].

Both IL-1 and TNF-alfa are produced after macrophage activation and produce an endothelial activation process leading to an increase of adhesion molecules expression, secretion of other cytokines, growth factors, ecosanoid production, nitric oxide and increased endothelial thrombogenicity. Both cytokines produce fibroblast activation leading to proliferation and augmented extracellular matrix synthesis. IL-1 and TNF-alfa produce stimulation of the acute phase systemic manifestation including somnolence, fever, changes in liver protein synthesis, changes in metabolism (caquexia), PMN migration, ACTH liberation and augmented steroid liberation [39].

Other effects of resveratrol upon on the liver can be the inhibition of nitric oxide production by the Kupffer cells [5] and antioxidant action similar to that of other substances such as S-adenosylmethionine [40].

## Conclusion

Resveratrol reduces mortality and liver damage produced by alcohol in mice. If our findings are confirmed by further research, resveratrol could be administered to patients with chronic alcoholism to reduce the mortality and liver damage associated with alcohol abuse. It could even be prophylactically added to alcoholic beverages, in a way similar (10 mg/L of any alcoholic beverage), similar to how chlorine is added to water to prevent infections.

## Competing interests

The author(s) declare that they have no competing interests.

## Authors' contributions

LB and MG-B conceived the idea and study design, conducted the analysis, drafted the manuscript; and in the decision to submit the manuscript for publication. JV, VG, MF, MG-S, ML, EH, ME-E, CS, JA participated in the collection, analysis, interpretation of data and helped to draft the manuscript. All authors read and approved the final manuscript. Not obtained funding.

## Pre-publication history

The pre-publication history for this paper can be accessed here:
